# Quinacridone-Diketopyrrolopyrrole-Based Polymers for Organic Field-Effect Transistors

**DOI:** 10.3390/ma6031061

**Published:** 2013-03-18

**Authors:** Masahiro Akita, Itaru Osaka, Kazuo Takimiya

**Affiliations:** 1 Department of Applied Chemistry, Graduate School of Engineering, Hiroshima University, 1-4-1 Kagamiyama, Higashi-Hiroshima, Hiroshima 739-8527, Japan; E-Mail: m112215@hiroshima-u.ac.jp; 2 RIKEN Center for Emergent Matter Science, Wako, Saitama 351-0198, Japan; E-Mail: takimiya@riken.jp

**Keywords:** semiconducting polymers, pigment, quinacridone, diketopyrrolopyrrole, organic field-effect transistors

## Abstract

Incorporation of pigment or dye molecules as building units is of great interest in the development of semiconducting polymers, due to their strong intermolecular interactions arising from the strong local dipoles in the unit structure, which would facilitate the charge transport property. In this paper, semiconducting polymers based on well-known pigments, namely, quinacridone and diketopyrrolopyrrole, are synthesized and characterized. The π-stacking distances are found to be 3.5–3.8 Å, which is fairly narrow for semiconducting polymers, indicating that they possess strong intermolecular interactions. Interestingly, polymer orientation is influenced by the composition of alkyl side chains. While the edge-on orientation is observed when the linear alkyl groups are introduced for all the side chains, the face-on orientation is observed when the branched alkyl groups are introduced either in the quinacridone or diketopyrrolopyrrole unit. It is found that the electronic structure of the present polymers is mostly affected by that of the diketopyrrolopyrrole unit, as evidenced by the absorption spectra and computation. Although the field-effect mobility of the polymers is modest,* i.e.*, in the order of 10^−4^–10^−3^ cm^2^/Vs, these findings could be important information for the development of semiconducting polymers.

## 1. Introduction

Owing to the excellent electrical and optoelectronic properties, semiconducting polymers have been attracting extensive attention in solution-processed organic electronics, such as field-effect transistors (OFETs) and photovoltaics (OPVs), which can offer large-area and flexible devices of next generation [1,2]. In particular, OFETs are an important fundamental component for such devices [3,4]. The main charge transport path in the semiconducting polymer films is interchain π-orbital overlaps of face-to-face π-stacked backbones, and thus enhancement of the intermolecular π−π interaction is crucial for the improvement of OFET performances [5].

While incorporation of a π-extended fused ring [6,7,8,9] or both electron rich (donor) and electron poor (acceptor) rings into the polymer backbone [10,11,12] have been successful approaches for the development of strong π–π interaction, use of a dye/pigment molecule, such as diketopyrrolopyrrole (DPP) [12,13,14], perylenedicarboximide (PDI) [15,16,17], naphthalenedicarboximide (NDI) [17,18,19], and isoindigo (IID) [20,21] is also beneficial. This is apparently because that the local dipoles across the electron donating nitrogen to the electron withdrawing carbonyl group in the molecules, which may be called “internal donor–acceptor system”, can offer strong intermolecular interactions.

**Figure 1:**
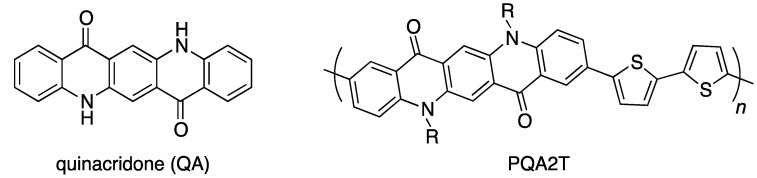
Chemical structure of quinacridone and a quinacridone-based semiconducting polymer.

Recently, we reported that the semiconducting polymers based on quinacridone (QA), a well-known red-violet pigment (Figure 1) [22], have strong π–π interactions with an interchain distance of 3.6 Å and exhibit relatively high hole mobilities of 0.2 cm^2^/Vs in OFETs [23]. This reveals that QA is a good building unit for semiconducting polymers. As QA is a p-type molecule and thus is a donor unit, combining with an acceptor dye unit can offer stronger intermolecular interactions due to the enhanced donor–acceptor system throughout the main chain, which could be useful for organic devices. Here, we report the synthesis, properties, structures, and OFET performances of new semiconducting polymers having QA and DPP as the building units.

## 2. Results and Discussion

**Scheme 1:**
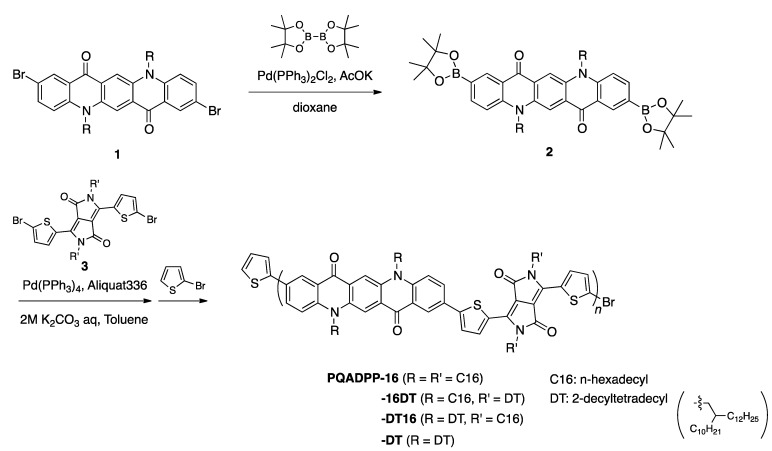
Synthesis and chemical structure of the polymers based on quinacridone (QA) and diketopyrrolopyrrole (DPP).

Scheme 1 shows the synthesis of QA-DPP polymers. 2,9-Dibromo*-N*,*N*-dialkylquino[2,3-*b*]acridine-7,14-dione (**1**) [24] was reacted with bis(pinacolato)diboron in the presence of Pd(PPh_3_)_2_Cl_2_ to afford 5,12-dialkyl-2,9-bis(4,4,5,5-tetramethyl-1,3,2-dioxaborolan-2-yl)quinolino[2,3-*b*]acridine-7,14(5*H*,12*H*)-dione (**2**) as the comonomer. **2** was then copolymerized with 3,6-bis-(5-bromo-thiophen-2-yl)-*N*,*N′*-bis(alkyl)-1,4-dioxopyrrolo[3,4-*c*]pyrrole (**3**) via the Suzuki-Miyaura coupling reaction, yielding the desired polymers. The alkyl groups introduced in **2** and **3** are *n*-hexadecyl (C16) and 2-decyltetradecyl (DT), and thus four polymers with different side chain combination were prepared (PQADPP-16, -16DT, -DT16, and DT). Molecular weight of the polymers is summarized in Table 1. While PQADPP-16DT, -DT16, and DT are soluble in chloroform, PQADPP-16 is only soluble in hot chlorobenzene or *o*-dichlorobenzene, which is likely as a result of the reduced solubility due to the introduction of linear alkyl groups in both the QA and DPP units. Whereas relatively high molecular weights of *M*_n_ > 15 kDa are obtained for PQADPP-16DT, -DT16, and -DT, PQADPP-16 gives low molecular weight of *M*_n_ = 7.4 kDa. This is probably due to the low solubility of the polymer as evidenced by the fact that the polymer had precipitated during the polymerization.

**Table 1:** Chemical properties of the polymers.

Polymer	*M*_n_ (kDa) ^a^	*M*_w_ (kDa)^ a^	PDI^ a^	DP_n_^ a^	*λ*_max_ (nm)^ b^		*E*_HOMO_ (eV)^ c^	µ (cm^2^/Vs) ^d^
					solution	film		
PQADPP-16	7.4	10.5	1.4	4.9	645, 704	653, 710	–5.2	6.8 × 10^−3^
PQADPP-16DT	28.1	91.7	3.3	16.2	624, 688	640, 696	–5.2	5.3 × 10^−4^
PQADPP-DT16	19.4	34.7	1.8	11.2	627, 689	642, 708	–5.2	2.3 × 10^−4^
PQADPP-DT	14.8	59.4	3.3	7.6	623, 686	630, 692	–5.2	N.D.

^a^ *M*_n_ = number-average molecular weight, *M*_w_ = weight-average molecular weight, PDI = polydispersity index, DP_n_ = degree of polymerization; ^b^ absorption maxima; ^c^ HOMO energy levels evaluated by photoelectron spectroscopy; ^d^ hole-mobilities calculated in the saturation regime (*V*_DS_ = −60 V).

**Figure 2:**
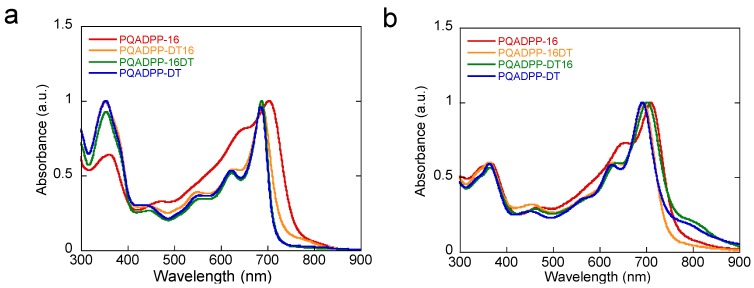
UV-Vis absorption spectra of the polymers in chlorobenzene solution (**a**) and in thin film (**b**).

Photoelectron spectroscopy (PESA) revealed that the HOMO level (*E*_HOMO_) of the polymers is ca. −5.2 eV. Figure 2 depicts the UV-Vis absorption spectra of the polymers in the chlorobenzene solution and in the film. Note that the spectrum for PQADPP-16 in the solution was measured at 100 °C, due to its low solubility. All polymers show similar spectra with the absorption maxima (*λ*_max_) and absorption edge (*λ*_edge_) of around 690 ~ 700 nm and 720 ~ 750 nm, respectively, in the solution. The *λ*_max_ slightly red-shifts by ca. 10 nm in the thin film. These *λ*_max_ in the present polymers are far red-shifted as compared to PQA2T, *λ*_max_ ≈ 470 nm and *λ*_edge_ ≈ 600 nm, but blue-shifted as compared to the common DPP-based polymers, e.g., for PDQT, *λ*_max_ ≈ 790 nm and *λ*_edge_ ≈ 900 nm.

**Figure 3:**
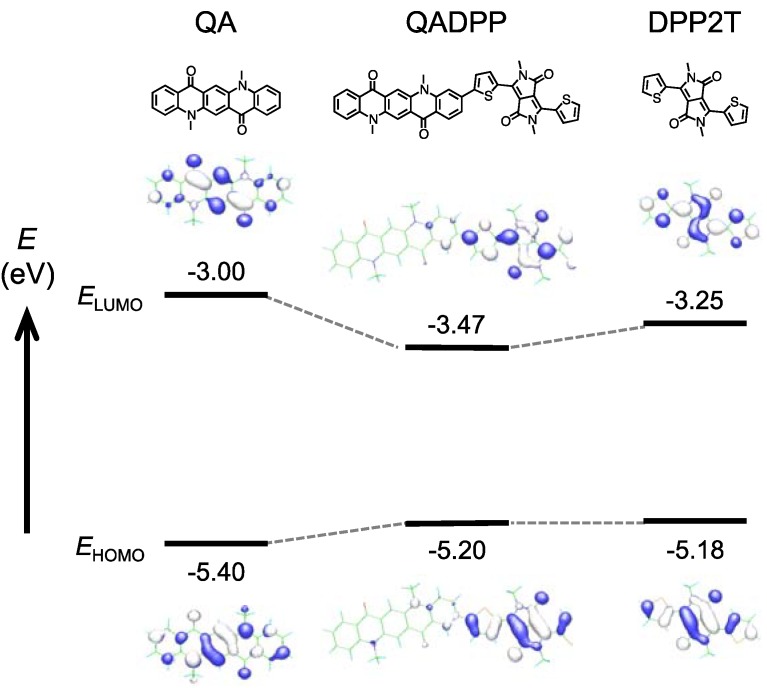
Computation (DFT at B3LYP/6-31(d) level) of the HOMO and LUMO geometry of quinacridone (QA, left), polymer repeat unit (QADPP, middle), and dithienyl diketopyrrolopyrrole (DPP2T), in which the methyl group is used as the substituents. HOMO (*E*_HOMO_) and LUMO levels (*E*_LUMO_) for each compound are also shown. *E*_HOMO_ and *E*_LUMO_ for QA and DPP2T were determined by electrochemistry. *E*_HOMO_ denoted as QADPP is that of the polymer (PQADPP-16DT) obtained by photoelectron spectroscopy in air (PESA), and *E*_LUMO_ is determined by the addition of optical bandgap to *E*_HOMO_.

Figure 3 shows the computation (DFT at B3LYP/6-31(d) level) of the HOMO and LUMO geometry of quinacridone (QA, left), polymer repeat unit (QADPP, middle), and dithienyl diketopyrrolopyrrole (DPP2T). *E*_HOMO_ and LUMO level (*E*_LUMO_) for each compound are also shown. *E*_HOMO_ and *E*_LUMO_ for QA and DPP2T were determined by electrochemistry. *E*_HOMO_ and *E*_LUMO_ used for QADPP are those of the polymer (PQADPP-16DT), in which *E*_LUMO_ is determined by the addition of the optical bandgap calculated from the absorption onset (1.73 eV) to *E*_HOMO_ (−5.2 eV). It is interesting to note that although HOMOs and LUMOs of both QA and DPP2T are distributed along the molecule long axes, those of QADPP localizes only on the DPP unit. It is likely that since *E*_HOMO_ and *E*_LUMO_ of DPP2T are higher and lower than those of QA, respectively, as depicted in Figure 3, the electronic structure of DPP2T is dominant in that of QADPP. This speculation may be supported by the fact that both *E*_HOMO_ and *E*_LUMO_ of the polymers are close to those of DPP2T.

**Figure 4:**
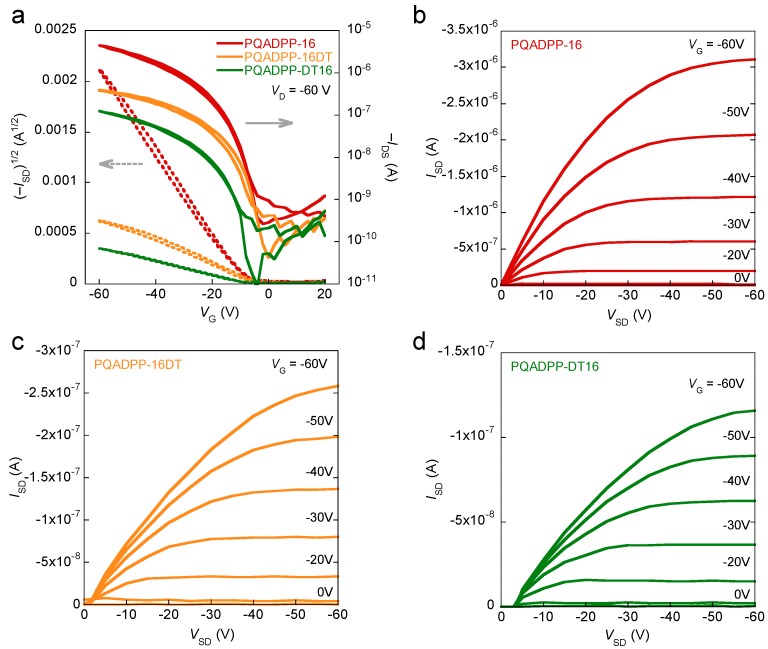
Transfer characteristics of OFETs based on PQADPP-16, -16DT, and -DT16 (**a**) and output characteristics of OFETs based on PQADPP-16 (**b**); -16DT (**c**); and -DT16 (**d**).

OFETs are fabricated using a bottom-gate top-contact configuration, in which the polymer solution was spin-coated on the hexamethyldisilazane (HMDS)-treated Si/SiO_2_ substrate and Au source and drain electrodes are vacuum-deposited after annealing the resulting polymer film at 150 °C for 30 min. Figure 4 shows the transfer curves and output curves of the polymer devices. While PQADPP-16, -16DT, -DT16 showed p-channel characteristics, PQADPP-DT was inactive. The maximum mobilities evaluated from the saturation regime are summarized in Table 1. Whereas modest mobilities, up to 6.8 × 10^−3^ cm^2^/Vs, are obtained for PQADPP-16, lower mobilities with the order of 10^−4^ cm^2^/Vs are obtained for PQADPP-16DT and -DT16. All mobilities obtained here are relatively low, and these results may relate to the localized HOMOs. Since the HOMO distribution play an important role in the charge (hole) transport both along the backbone and the intermolecular interaction, this localization might prevent the efficient hole transport. The variation of the mobility depending on the side chain composition can be well explained by the structural study using the two-dimensional grazing incidence X-ray diffraction (2D-GIXD) as described below.

**Figure 5:**
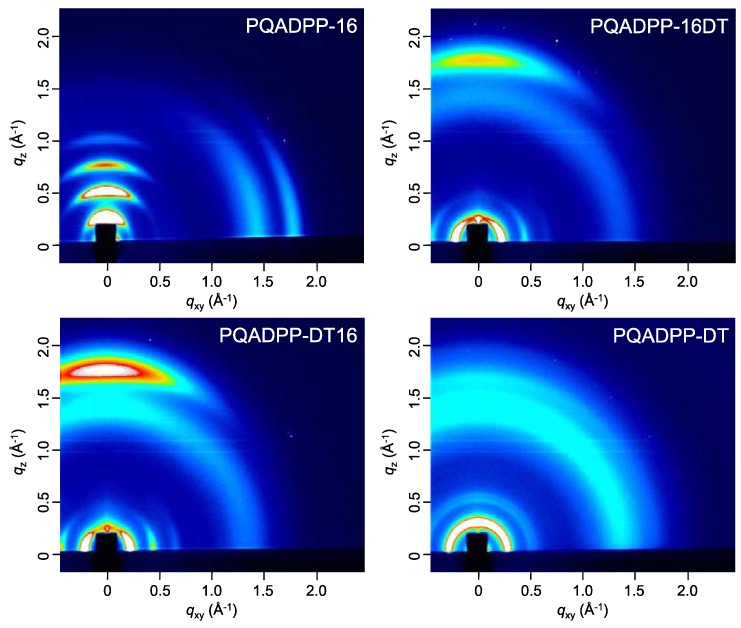
2D-GIXD patterns of the polymer films. GIXD: Grazing incidence X-ray diffraction.

Figure 5 shows the 2D-GIXD patterns of the polymer films after annealing at 150 °C. Interestingly, the polymer orientation is strongly dependent on the side chain composition. In the film of PQADPP-16, the diffractions corresponding to the lamellar and π–π stacking structures appear along the *q_z_* and *q_xy_* axes, respectively, indicating that the polymer chains are oriented in an edge-on manner. In the meantime, PQADPP-16DT and -DT16 exhibited the diffraction corresponding to the lamellar and π–π stacking structures along the *q_xy_* and *q_z_* axes, respectively, suggesting that the polymers form the face-on orientation. While the π–π stacking diffraction for PQADPP-16, -16DT, and -DT16 appears strong, that for PQADPP-DT is very weak. The π–π stacking distance (*d*_π_) for PQADPP-16 is found to be 3.5 Å, which is relatively narrow for semiconducting polymers. The *d*_π_ for PQADPP-16DT and -DT16, 3.6 Å, and -DT, 3.8 Å, are wider than that for PQADPP-16. These differences in the π–π stacking crystallinity can relate to the ratio of bulky branched group in the side chain. The long branched alkyl group may prevent the interchain interaction in the solid state, though it affords good solubility. Since the charge transport path in the semiconducting polymer films is primarily through the π–π interaction of face-to-face stacked polymer chains [5], such differences in the orientational motif and the π–π stacking is fairly consistent with the trend of the mobility.

## 3. Experimental Section

*Synthesis*. All chemicals and solvents are of reagent grade unless otherwise indicated. Chlorobenzene, and toluene were distilled prior to use. Molecular weights were determined by gel permeation chromatography (GPC) with a TOSOH HLC-8121GPC/HT at 140 °C using *o*-dichlorobenzene as a solvent and calibrated with polystyrene standards. 2,9-dibromo*-N*,*N*-dialkylquino[2,3-*b*]acridine-7,14-dione (1) [24] and 3,6-bis-(5-bromo-thiophen-2-yl)-*N*,*N′*-bis(alkyl)-1,4-dioxopyrrolo[3,4-*c*]pyrrole (3) [14] were synthesized according to the reported procedure.

*5,12-dihexadecyl-2,9-bis(4,4,5,5-tetramethyl-1,3,2-dioxaborolan-2-yl)quinolino[2,3-b]acridine-7,14(5H,12H)-dione (**2**)*. 1 (1.0 g, 1.09 mmol), dichlorobis(triphenylphosphine)dipalladium(II) (35 mg, 0.05 mmol), potassium acetate (642 mg, 2.64 mmol) was added to 25 mL of dioxane in a 100 mL 3-neck flask and purged with N_2_ for 30 min. After stirring the mixture for 1 h, bis(pinacolato)diboron (267 mg, 1.06 mmol) was added, which was then refluxed for 15 h. After cooling to room temperature, water was added and the aqueous layer was extracted with dichloromethane. The organic layer was washed with water then with brine, and then dried over anhydrous MgSO_4_. After removing the solvent by vacuum evaporation, the residue was purified by the purification with preparative GPC (chloroform) and recrystallized in ethyl acetate, which gave 2 as an orange solid (454 mg, 41% yield). ^1^H NMR (400 MHz, CDCl_3_): δ 9.05 (s, 2H), 8.78 (d, 2H,), 8.12 (dd, 2H, *J* = 7.8 Hz), 7.48 (dd, 2H, *J* = 8.8Hz), 4.53 (m, 4H), 2.00 (m, 4H), 1.39 (s, 24H), 1.50–1.20 (m, 52H), 0.90–0.86 (m, 12H). ^13^C NMR (400 MHz, CDCl_3_): ^13^C NMR (400 MHz, CDCl_3_): δ 178.3, 145.0, 140.0, 136.8, 136.5, 126.9, 121.0, 115.0, 114.9, 84.2, 50.1, 37.1, 32.3, 32.2, 32.1, 30.3, 30.0, 30.0, 30.0, 30.0, 29.9, 29.7, 29.7, 27.0, 25.3, 23.0, 23.0, 14.5. HRMS: Calcd for C_64_H_99_B_2_N_2_O_6_, [M + H]^+^: 1013.76838. Found: 1013.77008.

*5,12-di(2-decyltetradecyl)-2,9-bis(4,4,5,5-tetramethyl-1,3,2-dioxaborolan-2-yl)quinolino[2,3-b]acridine-7,14(5H,12H)-dione* (***2***). 1 (500 mg, 0.44 mmol), dichlorobis(triphenylphosphine)dipalladium(II) (15 mg, 0.02 mmol), potassium acetate (259 mg, 2.64 mmol) was added to 25 mL of dioxane in a 100 mL 3-neck flask and purged with N_2_ for 30 min. After stirring the mixture for 1 h, bis(pinacolato)diboron (267 mg, 1.06 mmol) was added, which was then refluxed for 15 h. After cooling to room temperature, water was added and the aqueous layer was extracted with dichloromethane. The organic layer was washed with water then with brine, and then dried over anhydrous MgSO_4_. After removing the solvent by vacuum evaporation, the residue was purified by preparative GPC (chloroform), which gave 2 as an orange solid (351 mg, 64% yield). ^1^H NMR (400 MHz, CDCl_3_): δ 9.08 (s, 2H), 8.87 (d, 2H),8.10 (dd, 2H, *J* = 8.8 Hz), 7.55 (dd, 2H, *J* = 8.7Hz), 4.53 (m, 4H), 2.25 (m, 2H), 1.38 (s, 24H), 1.50–1.20 (m, 80H), 0.90–0.84 (m, 12H).^13^C NMR (400 MHz, CDCl_3_): δ 178.3, 145.0, 140.0, 136.8, 136.5, 126.9, 121.0, 115.0, 114.9, 84.2, 50.1, 37.1, 32.3, 32.3, 32.1, 30.3, 30.0, 30.0, 29.9, 29.9, 29.8, 29.7, 29.7, 27.0, 23.0, 23.0, 14.5. HR-MS: Calcd for C_80_H_130_B_2_N_2_O_6_, [M + H]^+^: 1238.01878. Found: 1238.02075.

*General procedure for the polymerization.* A solution of 2 (0.10 mmol), 3 (0.10 mmol), Pd(PPh_3_)_4_ (2.3 mg, 2 mol %), and 1 drop of Aliquat 336 was added in 4 mL of toluene and 3 mL of aqueous K_2_CO_3_ (2M) were added to vial. The vial was then purged with argon and sealed. The resulting mixture was heated 180 °C for 40 min using a μ-wave reactor. After cooling to room temperature, the reaction mixture was precipitated into mixture of methanol (100 mL) 1 h at room temperature. 2-Bromothiophene (17 mg, 0.1 mmol) was then added and 180 °C for 40 min using a µ-wave reactor. Then the precipitated solid was subjected to sequential Soxhlet extraction with methanol and hexane to remove low molecular weight fractions (for PQADPP-DT, the sample was collected with hexane). The residue was extracted with chloroform and then precipitated in 200 mL of methanol to yield the desired sample as red solids (yield = 23%–92%).

*Measurements.* UV-Vis absorption spectra were measured using a Shimadzu UV-3100 spectrometer. HOMO level (*E*_HOMO_) was determined from the onset of photoelectron spectra measured by using a RIKEN KEIKI CO., LTD photoelectron spectrometer MODEL AC-2 in air. Cyclic voltammograms (CVs) were recorded on a BAS electrochemical analyzer, model 612D, in dichloromethane containing tetrabutylammonium hexafluorophosphate (Bu_4_NPF_6_, 0.1 M) as the supporting electrolyte at a scan rate of 100 mV/s. The counter and working electrodes were made of Pt, and the reference electrode was Ag/AgCl. All the potentials were calibrated with the standard ferrocene/ferrocenium redox couple (Fc/Fc^+^): E1/2 = +0.43 V measured under identical conditions). MO calculations were carried out with the DFT method using Gaussian 03 program package; Frisch, M.J. *et al.*, revision C.02; Gaussian, Inc., Wallingford, CT, 2004. Grazing incidence X-ray diffraction (GIXD) experiments were conducted at the SPring-8 on beamline BL19B2. The sample was irradiated at a fixed incident angle on the order of 0.12° through a HUBER diffractometer and the GIXD patterns were recorded with a 2-D image detector (PILATUS 300K). GIXD patterns were recorded with X-ray energy of 12.39 keV (λ = 1 Å).

*Device fabrication and characterizations.* OFET devices were fabricated in a “top-contact” configuration on heavily doped *n*^+^-Si (100) wafers with 200-nm-thick thermally grown SiO_2_ (*C_i_* = 17.3 nF cm^−2^). The Si/SiO_2_ substrates were carefully cleaned and then treated with hexamethyldisilazane (HMDS) to form a self-assembled monolayer (SAM), in which the silicon wafers were exposed to FDTS vapor at 100 °C in a closed desiccator for 3 h under nitrogen. Polymer layers were then spin-coated from warm (~80 °C) 3 g/L dichlorobenzene solution with 2500 rpm for 45 s, subsequently annealed at 150 °C for 30 min under nitrogen. On top of the polymer thin films, Au drain and source electrodes (thickness 80 nm) were deposited in vacuum through a shadow mask, where the drain-source channel length (*L*) and width (*W*) are 50 μm and ca. 1.5 mm, respectively. Current-voltage characteristics of the OFET devices were measured at room temperature in air with a Keithly 4200-SCS semiconductor characterization system. Field-effect mobilities were calculated in the saturation regime (*V*_SD_ = −60 V) of the *I*_SD_ using the following equation,

*I*_SD_ = (*WC*_i_/2*L*)µ(*V*_G_ − *V*_th_)^2^

where *C*_i_ is the capacitance of the SiO_2_ dielectric, *I*_SD_ is the source-drain current, and *V*_SD_,* V*_G_ and *V*_th_ are the source-drain, gate and threshold voltages, respectively. Current on/off ratios (*I*_on_*/I*_off_) were determined from the minimum current at around *V*_G_ = 0–20 V (*I*_off_) and the current at *V*_G_ = −60 V (*I*_on_). The mobility data were collected from more than 10 different devices.

## 4. Conclusions

We have synthesized and characterized semiconducting polymers based on QA and DPP pigment dyes. Although the transistor properties of these polymers were modest, some interesting information was found. The electronic structure of DPP is dominant in this system; *E*_HOMO_ and *E*_LUMO_ of the polymers are mostly affected by those of the DPP unit. Introduction of the two dye units in the polymer backbone leads to highly crystalline structure in the thin film with a close π-stacking distance of ~3.5 Å. The topology of the alkyl substituents significantly influence the orientation of the polymers, in which the polymer tends to orient in a edge-on manner when only the linear alkyl group is introduced, and in a face-on manner when, even partially, the long branched alkyl group is introduced. We believe that these results are of importance for the design of high-performance semiconducting polymers.
